# Supplementary material for: Dynamical Rearrangement of Human Epidermal Growth Factor Receptor 2 upon Antibody Binding: Effects on the Dimerization
Source: Biomolecules. 2019 Nov 5;9(11):706. doi: 10.3390/biom9110706 (PMC6920943; doi:10.3390/biom9110706)
Supplement: Supplementary file 1 [file biomolecules-09-00706-s001.pdf]

biomolecules

Dynamical rearrangement of human epidermal growth factor receptor 2 upon antibody binding: effects on the dimerization

Pedro R. Magalhães[1], Miguel Machuqueiro[1], José G. Almeida[2], André Melo[3], M. Natalia D. S. Cordeiro[3], Sandra Cabo Verde[4], Zeynep H. Gümüş[5], Irina S. Moreira[6], João D. G. Correia[4*], Rita Melo[4,6*]

[1] Centro de Química e Bioquímica and Departamento de Química e Bioquímica, Faculdade de Ciências, Universidade de Lisboa, 1749-016 Lisboa, Portugal, pedro.raf.magalhaes@gmail.com, machuque@ciencias.ulisboa.pt
[2] European Bioinformatics Institute, Cambridge CB10 1SD, United Kingdom, josegcpa@ebi.ac.uk
[3] REQUIMTE/LAQV, Faculdade de Ciências da Universidade do Porto, Departamento de Química e Bioquímica, Rua do Campo Alegre, 4169-007 Porto, Portugal, asmelo@fc.up.pt, ncordeir@fc.up.pt
[4] Centro de Ciências e Tecnologias Nucleares and Departamento de Engenharia e Ciências Nucleares, Instituto Superior Técnico, Universidade de Lisboa, CTN, Estrada Nacional 10 (km 139,7), 2695-066 Bobadela LRS, Portugal, ritamelo@ctn.tecnico.ulisboa.pt, jgalamba@ctn.tecnico.ulisboa.pt
[5] Department of Genetics and Genomics and Icahn Institute for Data Science and Genomic Technology, Icahn School of Medicine at Mount Sinai, New York, NY 10029, USA, zeynep.gumus@gmail.com
[6] CNC - Center for Neuroscience and Cell Biology; Rua Larga, FMUC, Polo I, 1ºandar, Universidade de Coimbra, 3004-517; Coimbra, Portugal, irina.moreira@cnc.uc.pt
Correspondence: jgalamba@ctn.tecnico.ulisboa.pt (J. D. G. Correia); ritamelo@ctn.tecnico.ulisboa.pt (R. Melo)., Tel.: 00351219946258

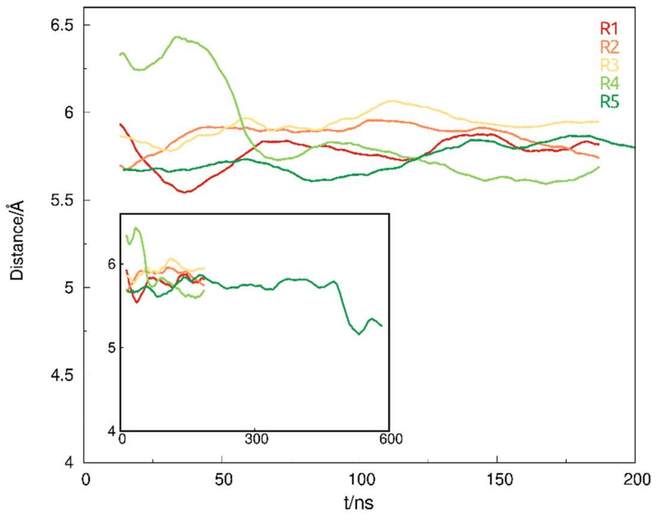

Figure S1. Time evolution of the COM distance between HER2 and F0178. The smaller plot shows the full length of the larger production run for this system. Data was smoothed using a floating average window of 10 ns.

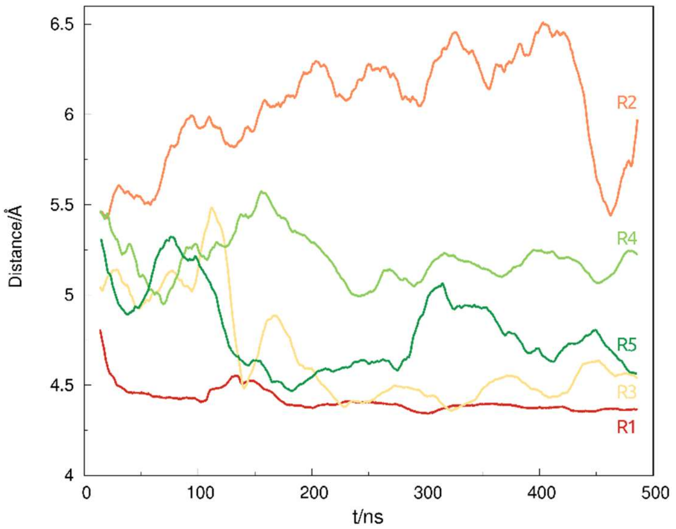

Figure S2. Time evolution of the COM distance between HER2 and ScFv. Data was smoothed using a floating average window of 10 ns.

biomolecules

MDPI

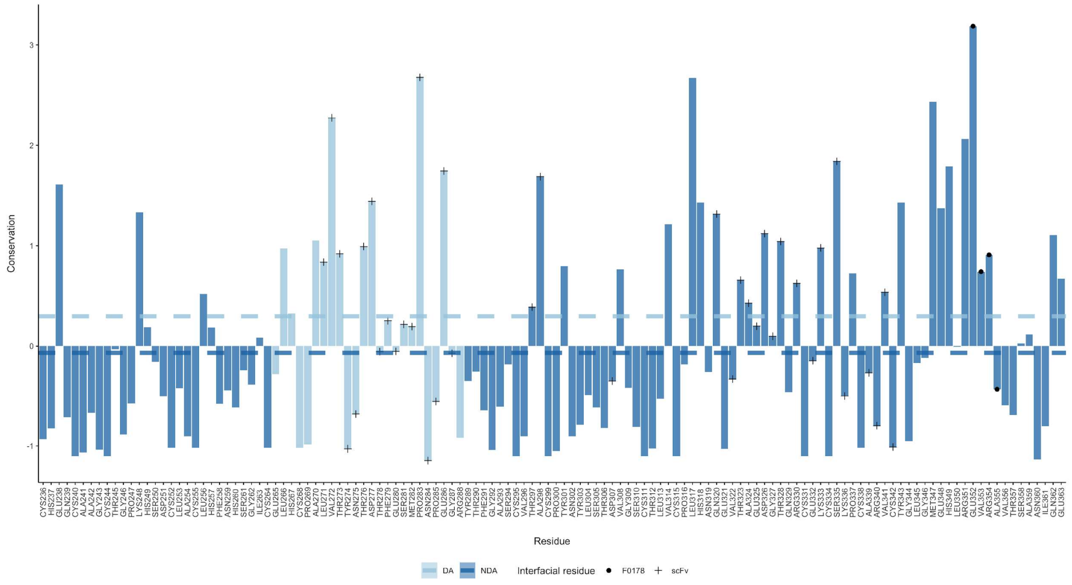

Figure S3. Normalised conservation scores of interfacial residues from ECDII-HER2 when coupled to F0178 (•) and scFv (+) antibody fragments. The light blue bars highlight the so-called "dimerization arm" residues. The light blue and dark blue dashed lines represent the average conservation score for dimerization arm and non-dimerization arm residues, respectively.

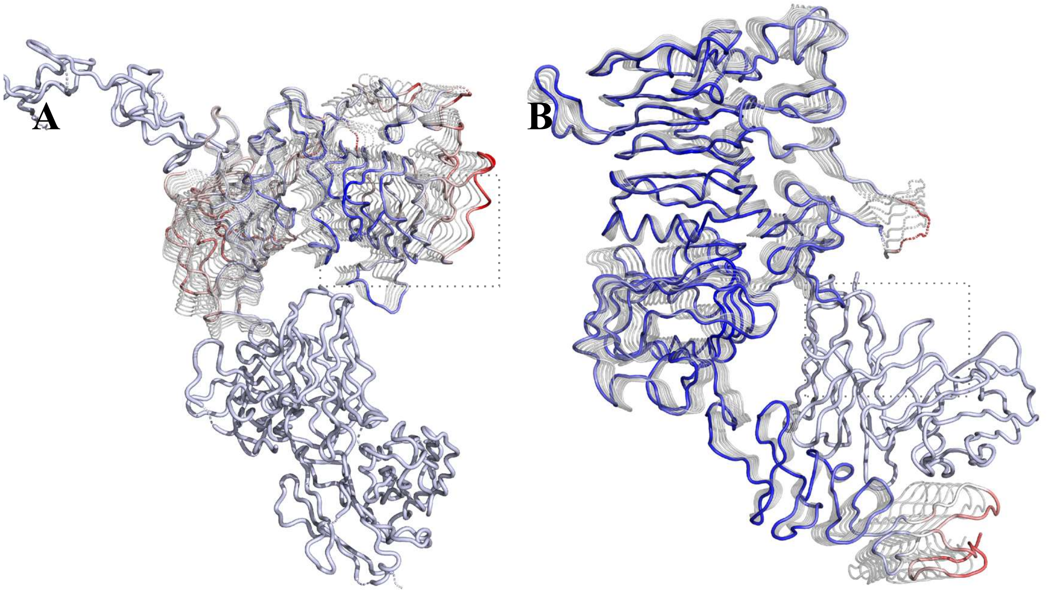

Figure S4. Interpolated structures along PC1: (A) F0178:HER2, (B) scFv:HER2. Colour scale from blue to red depict low to high atomic displacements. The dashed box highlights the "dimerization arm" region at ECDII.

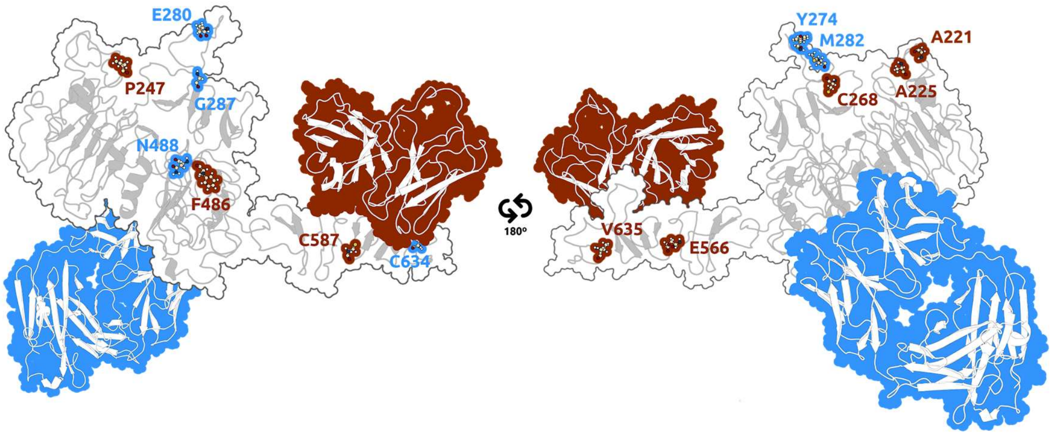

Figure S5. Location of the residues that showed a significant shift in SASA due to allosteric effects. The regions coloured in red show the residues in HER2 whose SASA was affected by the presence of ScFv, while the regions in blue show the residues in HER2 whose SASA was affected by F0178. Also shown are the initial positions of both partners (semi-transparent). The image on the right is the mirror-image of the one on the left. The secondary structure of the receptor and its partners is depicted as a cartoon, and the surface is also shown as a contour.

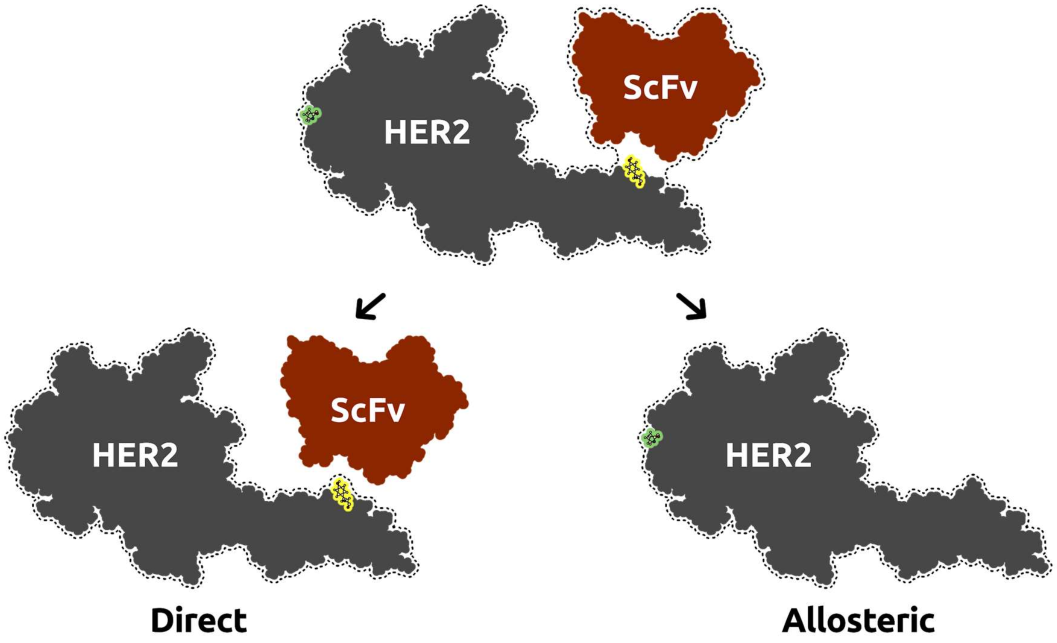

Figure S6. Simplified depiction of the SASA analysis. The dotted lines are a simplified depiction of the region used in the SASA calculation. Two specific residues coloured in green and yellow are also included. The residue in yellow is located in the interface region between HER2 and ScFv and should have a noticeable difference in SASA if ScFv is included or excluded from the calculation in the complex trajectory. The residue in green is located in a region where the presence of ScFv could only be felt indirectly when performing differences between the bound and unbound trajectories

biomolecules

Table S1. MD equilibrated regions for each system and replicate in nanoseconds.

	R1	R2	R3	R4	R5
**HER2**	40-200	50-200	50-200	20-200	--
**HER2:ScFv**	60-500	50-500	90-500	90-500	150-500
**HER2:F0178**	50-200	50-200	50-200	50-200	30-600
